# Diagnostic Value of Uterine Hemorrhage after the Menopause

**Published:** 1884-01

**Authors:** 


### Diagnostic Value of Uterine Hemorrhage After the Menopause.

During the course of a late clinical lecture on malignant disease of the cervix uteri, Dr. T. Gaillard Thomas stated, as an axiom in gynecology, that if a woman who has normally ceased to menstruate begins to have uterine hemorrhage, always suspect carcinoma. Not infrequently will you see in the medical journals the reports of cases begun to menstruate regularly again; but such accounts are altogether deceptive, and, if these cases could be followed out, it would be found, with scarcely a single exception, that the uterine flow was merely the indication of the presence of malignant disease. In other words, there is absolutely no such thing as the return of the menses when a woman has once reached the normal menopause. Not long since a patient of mine in the Woman's Hospital, who is sixty years of age, began to have a flowing from the uterus, and, as there was no indication of any external disease, I applied the curette to the endometrium and drew out some pulpy masses, which I sent to a well-known microscopist for examination. The report that I got from him was that the growth was not malignant in any respect, but simply a form of polypus. I am perfectly sure, however, that the microscopist is wrong, and for this reason: In the uterus of a woman of sixty, polypi never develop. The organ at that age is completely atrophied. Sometimes in women who have passed the menopause you will find uterine tumors which have all the appearance of fibroids. They are not by any means fibroids, however, but sarcomata.—*New York Medical Journal.*

